# Effects of raw and fermented rapeseed cake on ruminal fermentation, methane emission, and milk production in lactating dairy cows

**DOI:** 10.1016/j.anifeedsci.2023.115644

**Published:** 2023-06

**Authors:** Min Gao, Adam Cieślak, Haihao Huang, Maciej Gogulski, Daniel Petrič, Diāna Ruska, Amlan Kumar Patra, Mohamed El-Sherbiny, Małgorzata Szumacher-Strabel

**Affiliations:** a Department of Animal Nutrition, Poznań University of Life Sciences, Wołyńska 33, 60-637 Poznań, Poland; b Department of Preclinical Sciences and Infectious Diseases, Poznań University of Life Sciences, Wołyńska 35, 60-637 Poznań, Poland; c University Center for Veterinary Medicine, Poznań University of Life Sciences, Szydłowska 43, 60-637 Poznań, Poland; d Centre for Biosciences, Institute of Animal Physiology, Soltesovej 4-6, 040-01 Kosice, Slovak Republic; e Faculty of Agriculture, Institute of Animal Sciences, Latvia University of Life Sciences and Technologies, Liela Street 2, LV-3001 Jelgava, Latvia; f Department of Animal Nutrition, West Bengal University of Animal and Fishery Sciences, 37 K. B. Sarani, Kolkata, India; g American Institute for Goat Research, Langston University, Langston, OK 73050, USA; h Department of Dairy Science, National Research Centre, 33 Bohouth St., Dokki, Giza 12622, Egypt

**Keywords:** Fermented rapeseed cake, Enteric methane, Ruminal fermentation, Milk yield, Fatty acid profile

## Abstract

The objective of this study was to evaluate the effects of replacing raw rapeseed cake (RC) with fermented rapeseed cake (FRC) in the diet of dairy cows on methane (CH_4_) production, ruminal fermentation, and milk production, composition, and fatty acid composition. The Hohenheim gas test (exp. 1) was initially used to evaluate RC and FRC as substrates. Following batch fermentation, an in vitro study (exp. 2) was performed to assess the effects of replacing RC with FRC at 28.75, 57.5, 86.25, and 115 g/kg (FRC25, FRC50, FRC75, and FRC100) in the total mixed rations (TMR). Based on the in vitro results, the control TMR (115 g/kg dry matter (DM) of RC; CONRC) and experimental TMR (115 g/kg DM of FRC; FRC100) were chosen for an in vivo assessment. In exp. 3, four ruminally cannulated cows were used in a replicated 2 (group) × 2 (period) crossover design and fed the TMR ad libitum. In exp. 4, twenty multiparous Polish Holstein-Friesian cows in their mid-lactation (148 ± 26 d in milk) were used in a completely randomized design. The cows were fed a partial mixed ration without the RC and FRC, and the RC and FRC were supplied in a concentrate feeder at 2.65 kg/d/cow. The FRC100 markedly decreased CH_4_ production by 12% and archaeal population without adversely affecting nutrient digestibility. The molar proportion of propionate was increased, and the molar proportion of acetate and butyrate and acetate to propionate ratio were decreased by FRC100. No significant effects on milk production or composition, except an increase in milk urea concentration, were observed in cows fed FRC100. Milk C18:2 *cis*-9, *trans*-11 concentration was greater, and n-6 to n-3 fatty acid ratio was lower for FRC100 than CONRC. *In-situ* ruminal degradation of RC and FRC were explored using *in-sacco* techniques (exp. 5). The potential degradation and effective degradability of the DM, organic matter, and crude protein were significantly higher for FRC than RC. These results suggested that FRC could mitigate enteric CH_4_ production by decreasing archaeal abundances without adversely affecting milk production and ruminal fermentation in lactating cows.

## Highlights

• Fermentation of rapeseed cake (FRC) with 6-phytase expressed in *Pichia pastoris* increased crude protein content.
• FRC lowered ruminal acetate and increased propionate proportion.
• Dietary FRC increased beneficial fatty acid content in milk.
• Antimethanogenic impact of FRC was confirmed both in vitro and in vivo.

## 1 Introduction

Rapeseed production has become the largest source of oilseeds in the European Union, reaching a gross production of around 19 million tons in 2022, exceeding the gross production of sunflower and soybean seeds combined, making it the primary source of vegetable oil in Europe (Eurostat, 2022). Globally, rapeseed production is projected to be as high as 80.3 million tons in 2022, of which 30.7 and 37.9 million tons in the form of rapeseed oil and meal, respectively (USDA, 2022). The extraction of rapeseed oil by pressing rapeseeds results in the production of raw rapeseed cake (RC), a by-product known for its rich content of protein, fiber, and minerals, which makes it a potential protein source for animal nutrition (Bayat et al., 2021; Gao et al., 2022). Goiri et al. (2021) showed that feeding diets containing RC to lactating dairy cows increased milk production. It can be however expected that dietary RC may disturb ruminal fermentation. Gao et al. (2021) also reported that the inclusion of RC with high glucosinolates content in growing steers diet decreased ruminal ammonia concentration and CP digestibility without affecting total VFA concentration. Feeding RC reduced the total saturated fatty acid (SFA) profile and increased the total monounsaturated fatty acid in the rumen of dairy cows (Goiri et al., 2021); however, inconsistent results were observed in milk fatty acid (FA) composition. Kudrna and Marounek (2006) observed higher profiles of stearic acid, vaccenic acid, and conjugated linoleic acid (CLA) with no effect on the total polyunsaturated fatty acids (PUFA) profile in milk, whereas Goiri et al. (2021) reported no changes in most of the milk fatty acids including the CLA profile. Despite a few promising impacts of RC, it is recommended to use it in limited amounts because of the presence of antinutritional factors in it, such as glucosinolates, phytic acid, alkaloids, erucic acid, and tannins (Nega, 2018, Song et al., 2022).

The solid-state fermentation (SSF) process is one of the most recommended approaches to enrich the nutritional value of agricultural by-products mainly by degrading fiber (cellulose and lignin) and eliminating anti-nutritional factors (glucosinolates and phytic acid) (Chiang et al., 2010, Parmar et al., 2019, Song et al., 2022). Interestingly, incorporating SSF materials at a rate of 5–20% in both ruminant and non-ruminant diets may increase growth, production, and health status with a possible reduction in CH_4_ production and better management of the feeding costs (Parmar et al., 2019). The results reported by Shi et al. (2015) showed that SSF of RC with *Aspergillus niger* reduced glucosinolates levels by about 77% and improved CP content by approximately 23%. The increase in CP in FRC results from an increase in microbial protein production followed by a reduction in dry matter (DM) content at the expense of fermentable sugars during SSF (Olukomaiya et al., 2019). As for the elimination of the antinutritional factors, Drażbo et al. (2019) reported that SSF of RC with 6-phytase enzyme resulted in a ten times reduction of glucosinolates content (16.3 versus 1.66 µmole/g).

The effects of FRC on milk production, ruminal fermentation, and fatty acid profile, when fed to ruminants, have not been studied yet; however, the results published on other fermented by-products tested as animal feeds seem to be promising. The study conducted by Shi et al. (2015) reported that in vitro amino acid digestibility and total tract digestibility in growing pigs were improved by fermented RC (FRC) and impressively, it was comparable to the performance of pigs fed a corn-soybean meal diet. Another study on fermented soybean meal highlighted increased molar proportions of propionate and valerate when it replaced soybean meal in lactating dairy cows’ diets (Wang et al., 2021). According to Aires et al. (2009), the hydrolysis of glucosinolates produces various metabolites, including isothiocyanates, thiocyanates, nitriles, allylamine, benzylamine, and many indole compounds from indole glucosinolates, and these compounds exhibit antimicrobial activities against both Gram-positive and Gram-negative bacteria. Some studies also reported that isothiocyanates altered ruminal fermentation characteristics and decreased CH_4_ (Lila et al., 2003, Soliva et al., 2011) and this could be attributed to a direct effect on methanogens or a consequence of promoting propionate formation known to decrease CH_4_ production by competing for ruminal hydrogen utilization (Moss et al., 2000). A trial conducted by Amin et al. (2022) showed an increase in milk yield, protein, and fat yields with the inclusion of fermented soybean meal in Holstein cows’ diet and in the same study, fermented soybean meal also increased the acetate percentage and the acetate to propionate ratio. Our hypothesis was that substituting FRC produced through SSF with RC in dairy cows’ diets may increase nutrient digestibility, positively modulate ruminal fermentation, mitigate CH_4_ formation, and improve milk production. Therefore, the objective of this study was to evaluate the impact of FRC on (i) mitigating CH_4_ production and modulating ruminal fermentation using in vitro trials, (ii) ruminal fermentation, CH_4_ emission and milk production and composition using in vivo trials, and (iii) ruminal nutrient degradation kinetics using an in situ trial.

## 2 Materials and methods

All experiments were conducted following the guidelines of the Local Ethical Commission for Investigations on Animals (permit no. 50/2021) and performed following the National Ethical Commission for Animal Research (Ministry of Science and Higher Education, Poland).

### 2.1 Experiments and diets

There were five successive experiments conducted separately. The first phase consisted of two in vitro experiments using the Hohenheim gas test (experiment 1) and batch culture (experiment 2) techniques. Based on the first phase results, two in vivo experiments on cannulated dairy cows (experiment 3) and lactating dairy cows (experiment 4) under commercial farm conditions were conducted and finally, a rumen *in sacco* nutrient degradation experiment (experiment 5) was performed.

The experimental diet (Table 1) consisted of a homogeneous mixture of maize silage (354 g/kg of DM), lucerne silage (88 g/kg of DM), grass silage (83 g/kg of DM), beet pulp (111 g/kg of DM), brewer’s grain (111 g/kg of DM), wheat (97 g/kg of DM), RC (115 g/kg of DM), commercial concentrate (31 g/kg of DM), and a mineral mixture (10 g/kg of DM). The rapeseed variety used in the study was BIRDY, where the concentration of glucosinolates in the raw material was 17.05 µmol/g, and after the fermentation process, the glucosinolates concentration was reduced to 2.01 µmol/g. The content of glucosinolates (μmol/g feed) was determined using gas chromatography using silyl derivatives of desulfo-glucosinolates (Michalski et al., 1995; and PN ISO 9167–1:1999, 1999). The double-low glucosinolates RC and the FRC were purchased from HiProMine (Robakowo, Poland) that prepared the FRC following the procedure of Drażbo et al. (2019) and patent procedure No. 237575 (Józefiak et al., 2017). Briefly, RC was ground and thoroughly mixed with water at a ratio of 1:2 in plastic containers. Only the 6-phytase expressed in *Pichia pastoris* was supplemented to the ground RC in a ratio of 1:1000 on a dry weight basis and thoroughly mixed. The SSF was processed for 24 h at 30 °C under anaerobic conditions, and then the enzyme was deactivated at 70 °C within 15 min. The biomass was dried at 55 °C. The chemical composition of all the feedstuffs and diets is shown in Table 1. All the TMR diets were formulated using FeedExpert software (Rovecom, Hoogeveen, Netherlands) to meet the nutrient requirements of cows in their mid-lactation (36 kg milk/d).

**Table 1:** Ingredient and chemical composition of the experimental diets (n = 3).

Item^a^	RC^b^	FRC^c^	SEM^d^	P-value	CONRC^e^	FRC100^f^	SEM	P-value	PMR^g^
Ingredient composition, g/kg DM									
Maize silage	-	-	-	-	354	354	-	-	400
Lucerne silage	-	-	-	-	88	88	-	-	99
Grass silage	-	-	-	-	83	83	-	-	94
Beet pulp	-	-	-	-	111	111	-	-	125
Brewer’s grains	-	-	-	-	111	111	-	-	125
Wheat	-	-	-	-	97	97	-	-	110
**RC/FRC**	-	-	-	-	**115**	**115**	-	-	-
Concentrate	-	-	-	-	31	31	-	-	37
Mineral and vitamin premix^h^	-	-	-	-	10	10	-	-	10
Chemical composition, g/kg DM									
Organic matter	931	930	0.65	0.55	906	905	2.63	0.90	928
Ash	69	70	0.65	0.55	94	95	2.04	0.99	72
Crude protein	361	386	7.23	0.07	156	158	4.12	0.87	138
Neutral detergent fiber	248	271	6.56	0.06	356	358	3.81	0.86	456
Ether extract	103	95	2.53	0.08	26	25	1.38	0.72	32
Fatty acids (FA), g/100 g FA									
C14:0	0.12	0.14	0.006	0.08	0.60	0.63	0.02	0.73	0.61
C16:0	6.63	6.69	0.02	0.07	20.2	20.3	0.20	0.90	21.9
C18:0	1.79	1.78	0.012	0.92	2.51	2.51	0.20	0.99	3.61
C18:1, *cis*-9	53.6	56.1	0.75	0.08	22.3	25.7	1.00	0.11	17.1
C18:1, *cis*-11	6.17	5.94	0.06	0.07	2.22	1.93	0.102	0.29	1.02
C18:2, *cis*-9, *cis*-12	21.3	19.4	0.57	0.07	42.5	39.9	0.76	0.08	43
C18:3, *cis*-9, *cis*-12, *cis*-15	8.18	7.35	0.24	0.02	8.43	7.33	0.33	0.11	8.08
Sum of SFA	8.90	9.10	0.08	0.33	23.7	24	0.14	0.47	27.7
Sum of UFA	91.1	90.9	0.08	0.33	76.3	76	0.14	0.47	72.3
Sum of MUFA	61.3	63.8	0.74	0.07	25.2	28.6	1.00	0.11	19.6
Sum of PUFA	29.8	27.1	0.81	0.05	51.1	47.4	1.07	0.06	52.7
Sum of n-6 FA	21.4	19.4	0.57	0.08	42.5	39.9	0.76	0.08	43.8
Sum of n-3 FA	8.27	7.46	0.23	0.02	8.43	7.37	0.32	0.11	8.49
n-6/n-3 FA ratio	2.59	2.60	0.02	0.74	5.04	5.06	0.05	0.89	5.16

^2,3^The diet was used in experiment 1.

^5,6,7^The TMR/PMR diets with either RC or FRC as a protein source were used in experiments 2–5.

a DM: dry matter; SFA: saturated fatty acids; UFA: unsaturated fatty acids; MUFA: monounsaturated fatty acids; PUFA: polyunsaturated fatty acids.

b RC: raw rapeseed cake.

c FRC: fermented rapeseed cake.

d SEM: standard error of means.

e CONRC: total mixed ration with RC as a protein source.

f FRC100: total mixed ration with FRC as a protein source.

g PMR: partial mixed ration.

h Declared to contain (g/kg of DM) Na (123), Ca (100), Mg (45), P (42), K (20), S (18), Co (14), Cu (5.0), Zn (2.8), Mn (1.4), Fe (1.05), F (0.42), I (0.028), Se (0.018), biotin (0.008); (IU/kg), vitamin A (200,000), vitamin D3 (40,000), and vitamin E (1200).

#### 2.1.1 In vitro experiment using the Hohenheim gas test technique (Experiment 1)

The Hohenheim gas test technique (Menke and Steingass, 1988) was employed to study the effects of RC and FRC on gas production, ruminal fermentation characteristics, and CH_4_ production. RC or FRC was used as the sole substrate in the fermentation media to exclude other factors related to the diets. This study was performed in a 2 × 5 (treatment × glass syringe) experimental arrangement with three consecutive runs (on different days). In total, fifteen repetitions were tested for each feedstuff. Briefly, fresh ruminal fluid for inoculum was collected from three cannulated Polish Holstein-Friesian dairy cows (620 ± 25 kg body weight and second month of lactation) before morning feeding. The rumen inoculum donors were fed 21 kg DM of a total mixed ration (TMR) containing 115 g/kg diet of RC (DM basis) (CONRC) and supplied twice daily at 0600 h and 1800 h (Table 1). Ruminal fluid was filtered through a two-layered cheesecloth into two Schott–Duran bottles maintained at 39 °C under anaerobic conditions and immediately transported to the laboratory. Standard artificial buffer solution contained 292 mg K_2_HPO_4_·3 H_2_O, 240 mg KH_2_PO_4_, 480 mg (NH_4_)_2_SO_4_, 480 mg NaCl, 100 mg MgSO_4_·7 H_2_O, 64 mg CaCl_2_·2 H_2_O, 4 mg Na_2_CO_3_, and 600 mg cysteine hydrochloride per liter. Ruminal fluid from the three animals was mixed on an equal volume basis and then combined with buffer solution in a 1:4 (v/v) ratio. The buffer solution was prepared according to Bryszak et al. (2019) protocol, with some modifications. Briefly, 20 mL of buffered ruminal fluid was transferred to calibrated 100-mL Hohenheim glass syringes (Haberle LaborTechnik, Lonsee-Ettlenschieß, Germany) containing 200 mg DM of feeds to be tested. The syringes were incubated for 24 h at 39 °C under anaerobic conditions and an atmospheric pressure of 101,325 Pa. Gas production was recorded at two-hour intervals. After 24 h, CH_4_ concentration in the headspace gas was analyzed, and buffered ruminal fluid samples were collected for analysis of ammonia, volatile fatty acids (VFA) and fatty acids (FA), protozoa, and methanogen populations. For the protozoa counts, 1 mL of the ruminal fluid was preserved with 6 mL of 8% formaldehyde solution (w/w), and stored at 4^◦^C in a refrigerator until analysis. Rumen fluid (1.5 mL) for methanogens analysis was shock-frozen in liquid nitrogen and samples were stored at − 80 °C until analysis. Samples collected for ammonia, VFA, and FA analysis were stored at − 20 °C.

#### 2.1.2 In vitro experiment using batch culture (Experiment 2)

This experiment was performed following the procedure of Bryszak et al. (2019). As described above, the rumen inoculum was obtained from three ruminal cannulated Polish Holstein-Friesian dairy cows. The 125-mL glass incubation bottles (Midland Scientific, Omaha, NE) containing 400 mg (DM basis per bottle) TMR substrates were incubated with 40 mL buffer solution (the same as used in Experiment 1). There were five treatment groups: a control group (CONRC) containing TMR, which included a 115 g/kg diet of RC (DM basis), and four experimental groups in which the RC in TMR was replaced with 28.75 (FRC25), 57.5 (FRC50), 86.25 (FRC75), and 115 (FRC100) g/kg diet of FRC (DM basis) at 25%, 50%, 75% and 100% levels. The TMR used in the CONRC group was the same as the control diet, which was used in the in vivo cannulated dairy cows experiment (Table 1). The experimental group TMR (FRC25, FRC50, and FRC75) had a chemical composition similar to CONRC and FRC100. Gaseous CO_2_ was added to each bottle containing buffered ruminal fluid and substrates. The bottles were closed with rubber stoppers, sealed with aluminum covers, and kept for 24 h in an incubator (Galaxy170R, Eppendorf North America, Hauppauge, NY) at a temperature of 39 °C. The batch culture experiment had five bottles in each group (5 groups × 5 bottles) completed in three consecutive runs (on different days). Fifteen repetitions for each substrate were also tested in this experiment. After 24 h of incubation, CH_4_ production and digestibility were measured.

#### 2.1.3 In vivo experiment using cannulated dairy cows (Experiment 3)

The experiment with cannulated dairy cows was based on batch culture study results demonstrating that the most significant reduction of CH_4_ emission and increases in vitro DM degradability (IVDMD) and total VFA concentration occurred in the FRC100 diet. The experiment was conducted according to the procedure used by Bryszak et al. (2019). Four ruminal cannulated Polish Holstein-Friesian cows (multiparous, 630 ± 30 kg body weight, 33.5 ± 1.29 kg/d milk production and fifth to sixth month of lactation) fitted with rumen cannulas (2 C, 4 in.; Bar Diamond, Parma, Idaho, USA) were allocated to two dietary groups (CONRC and FRC100; n = 4) in a replicated 2 (group) × 2 (period) crossover design. Each period lasted 30 d, with a 21-d adaptation and 9-d sampling period (three days of rumen fluid collection and six days for gas analysis). The cows were allowed to adapt to the respiratory chambers during an adaptation period (from d 5 to d 21). The control group was fed the TMR – CONRC with RC (115 g/kg diet) as a protein source (Table 1). In the experimental group, cows were fed the TMR – FRC100 with FRC (115 g/kg diet) as a protein source. All ingredients composition of the TMR was the same as the TMR used for the batch culture study. Feeds were provided twice daily at 0600 h and 1800 h. The cows had free access to water ad libitum. During the sampling period, 600-mL samples of ruminal fluid were collected from three locations in the midventral sac of the rumen just before (0 h) feeding and 3 h and 6 h after morning feeding (Grummer et al., 1993). Additionally, 3 h after morning feeding, the samples for determining total methanogen and rumen bacteria (species and genera) populations were collected. The DM intake (DMI) was measured daily for 4 d per period (d 22–25 per period) by weighing the individual amounts of feeds offered and leftovers in the feeding box. Total feces from individual cows were recorded and weighed during the DMI sampling days to determine the total-tract digestibility coefficients. Feces subsamples (50 g/kg, w/w) were prepared and stored at − 20 °C for DM, organic matter (OM), and CP analysis. Samples of RC, FRC, and fresh TMR (CONRC and FRC100) were collected 3 times a week from the farm and immediately delivered to the laboratory to perform chemical analyses.

#### 2.1.4 In vivo experiment using commercial dairy cows (Experiment 4)

The results obtained from the in vitro studies and the in vivo cannulated cows experiments showed lower CH_4_ emission and higher nutrient degradability in FRC diets compared with the RC diet. Hence, the maximum FRC dose was used in the commercial dairy cow experiment (FRC100; Table 1; 115 g/kg DM of TMR). Twenty multiparous Polish Holstein-Friesian cows [640 ± 31 kg body weight, 2.6 ± 0.32 parity, 148 ± 26 d in milk, and 35 ± 2.4 kg/d milk production; (mean ± SD)] were used in a completely randomized design with a 21-d adaptation and a 6-d sampling periods (a total of 27 d experimental period). The cows were randomly allocated to two dietary groups (10 cows in each group) - CONRC and FRC100 and kept separately into two groups in two pens (dedicated area of a barn). The cows were fed a partial mixed ration (PMR; Table 1), where RC or FRC (115 g/kg DM) was taken from the previously used TMR, and the feed registration was performed for each group. The RC or FRC was supplied at 2.65 kg/d/cow using a computer-controlled feeder station (De Laval, type FP 204, Tumba, Sweden) where cows were adapted to the concentrates. During the first 8 d of each adaptation stage, the cows were fed PMR ad libitum. From day 9 onwards, the feed was restricted to 95% of the average daily voluntary DMI, based on days 3–8 of the cows assigned to each treatment to avoid the confounding effects of DMI. Finally, DMI was maintained at 23.2 ± 0.7 kg/d during the experiment. Clean water was available ad libitum. Cows were milked twice daily at 0530 h and 1730 h, and daily milk production was measured using a milk meter (WB Ezi-Test Meter 33 kg; Tru-Test, Manukau, New Zealand). During the last three sampling days, milk samples (ca. 60 mL) were collected for chemical analysis: one part was used for the analysis of milk basic constituents, the second part (7 mL) was stored at − 20 °C for FA analysis, and the rest (10 mL) was immediately stored in liquid nitrogen for gene expression analysis. CH_4_ concentration was continuously measured for the whole sampling period (6 days).

#### 2.1.5 In sacco ruminal degradability (Experiment 5)

The aim of this in situ study was to examine the potential degradation (PD) and effective rumen degradability (ED) of DM, OM, and CP of RC or FRC (n = 6; 3 repetitions × 2 runs). Two multiparous Polish Holstein-Friesian cows (620 ± 15 kg body weight) fitted with rumen cannulas (2 C, 4 in., Bar Diamond, Parma, Idaho, USA) were used. The cows were fed twice daily at 0600 h and 1800 h with TMR, where RC was used (Table 1). Clean water was available ad libitum. The cows were adapted to the diet for three weeks before the in situ incubation. RC was used as a control feed to compare the FRC as an experimental feed for its degradability. Samples of 3 g DM were placed in each Dacron bag (6 × 13 cm; pore size 50 µm) and sealed with insoluble surgical sutures approximately 1 cm below the upper edge. These bags were then placed in a large mesh (35 cm × 60 cm) with fine sieves (0.5 cm × 0.5 cm), allowing the ruminal fluid to freely access to feed samples placed in the bags. Weights were used for better mesh placement with the incubated bags in the rumen content. Bags were placed in the ventral sac of the rumen for 0, 2, 4, 8, 16, 24, 48, and 72 h. Bags used for 0 h time disappearance were soaked in distilled water at 39 °C for 15 min. For each sample and each time of incubation, three (0, 2, 4, 8, and 16 h) or six (24, 48, and 72 h) bags were incubated in each cow (total 33 bags/feed). All samples were incubated in the rumen of cows in 2 runs (total 66 bags/feed). After the appropriate incubation time, the bags were removed from the rumen, and the fermentation of the material in the bags was inhibited by placing the bags in cold water. The bags were then rinsed with cold water until clean water was obtained and stored in a freezer at − 20 °C until dried. The bags were dried at 60 °C for 48 h using a dryer with forced air circulation. The dried materials were then weighed to obtain the mass of the materials after incubation. After weighing, the samples were ground to pass through a 1-mm sieve using a ZM 200 mill (Retsch, Düsseldorf, Germany) and prepared for further analysis. The PD and ED of DM, OM, and CP were calculated as described below.

### 2.2 Data collection and laboratory analysis

#### 2.2.1 Chemical analysis and fatty acid profile

The chemical composition of RC, FRC, PMR, and TMR was analyzed following AOAC methods (Horwitz and Latimer, 2007) for the determination of DM (method no. 934.01, drying at 103 °C for 6 h) and ash (method no. 942.05, burning at 550 °C for 3 h). Crude protein (N-content; method no. 976.05, constant factor for CP: 6.25) content was determined using a Kjel-Foss Automatic 16210 analyzer (Foss Electric, Hillerød, Denmark), and ether extract without acid hydrolysis (EE, method no. 920.39) was determined with a Soxhlet System HT analyzer (FOSS, Hillerød, Denmark). Ash subtracted from DM represented the OM content. The neutral detergent fiber was derived with the addition of amylase and sodium sulfite without residual ash (aNDFom) (Van Soest et al., 1991). The chemical composition of the residue substrate after the *in sacco* incubation was determined using the above methods for DM, OM, and CP. The FA profile of feeds, ruminal fluid, and milk samples were examined and identified as described previously (Bryszak et al., 2019).

#### 2.2.2 Ruminal fermentation analysis

The pH of the ruminal samples from in vitro fermentation (experiments 1 and 2) and the in vivo experiment (experiment 3) was measured immediately after collection of samples using a pH meter (Type CP-104, Elmetron, Zabrze, Poland). Ammonia concentration was determined using the colorimetric Nessler method (Bryszak et al., 2019). The loss in weight of the incubated substrate DM after correction for the DM residue in the blank was taken as IVDMD. *In vitro* organic matter degradability (IVOMD) was determined as the loss in weight of the incubated feed OM. A gas chromatograph (GC Varian CP 3380, Sugarland, TX) with a flame ionization detector was used to determine VFA concentrations following the protocol described by Bryszak et al. (2019).

#### 2.2.3 Total gas and methane production analysis

In experiments 1 and 2, the total gas volume was recorded, and CH_4_ production 24 h after incubation was measured using a GC (SRI Peak Simple model 310 Alltech, PA), according to the procedure used by Bryszak et al. (2020). Gas production was determined by the piston displacement of calibrated 100-mL Hohenheim glass syringes (Haberle LaborTechnik, Lonsee-Ettlenschieß, Germany), which were connected to the serum flasks. During the gas measurement, the temperature was 39 °C, and the atmospheric pressure of 101,325 Pa. For CH_4_ concentration determination, 500 μl gas was sampled from Hohenheim glass syringes (kept at room temperature of 20 °C and atmospheric pressure of 101,325 Pa) in a gastight syringe (GASTIGHT® Syringes, Hamilton Bonaduz AG, Switzerland) and injected into GC. The CH_4_ production was calculated in mmol using the following equation: n = pV/r'MT; where p = pressure (Pa), V = volume (m^3^), rM = 0.519 (8.314/16.04), T = temperature 293.15 K. In experiment 3, from d 25–30, two cannulated cows were transferred individually into two respiratory chambers (W × L × H: 300 × 400 × 220 cm; SPA System, Wrocław, Poland). Two nondispersive infrared spectroscopy analyzers operating in the near-infrared spectrum (detector 1210 Gfx, Servomex 4100, Servomex, UK) were used to determine CH_4_ and CO_2_ concentrations. During the sampling days, the time of milking (approximately 30 min) in the morning and evening feedings was excluded from the gas emission calculations. Individual cows were transferred into a respiratory chamber to determine the direct CH_4_ emission for 23.5 h consecutively for 3 d. Cows were restrained within the chambers by a neck yoke on a dedicated platform (180 × 126 cm) covered with a rubber mat and had free access to fresh water and salt block. Measurements were taken every 3-second intervals. Two measuring channels were applied: the concentration of CO_2_ in the range of 0–2.5% (0–48,450 mg/m^3^) and the CH_4_ concentration in the range of 0–1000 ppm (0 −706 mg/m^3^). Samples were collected and ducted to the analyzer via a polyethylene tube with an 8 mm diameter and the sampling rate was 0.6 L/min. The ventilation rate, relative humidity, and temperature in each respiratory chamber were maintained at about 9.8 m^3^/min, 60%, and 20 °C, respectively, with the help of a air-conditioning system. Total airflow was measured using in-line hot wire anemometers that were validated by daily measurements made with an externally calibrated anemometer (Testo 417; Testo Limited, Poland). The exhausted air was replaced with fresh air drawn through a large-diameter PVC duct of 30 m length from the respiratory chamber. The accuracy of the chambers was checked before starting each period with a known volume release of ultrapure (>99.9%) CH_4_ (Linde Gases Pty. Ltd., Thomastown, Victoria, Australia). Before the start of the experiment, gas recoveries were also measured by releasing CO_2_ at a constant rate into each chamber. The mean recovery was 98% (SEM = 3·0), which was adjusted for actual volume calculations. The analyzers were calibrated using calibration gasses before starting the experiments: nitrogen (99.999% purity), 1210 ppm CH_4_ in nitrogen, and 4680 ppm CO_2_ in nitrogen. The analyzer was equipped with a 0.17 L cuvette with a 540 mm optical track length for CH_4_ and a 0.012 L cuvette with a 154 mm optical track length for CO_2_. In experiment 4, during the six days of the collection period, CH_4_ concentration was continuously measured during the feeding of RC or FRC at a computer-controlled feeder station using the same type of detectors as in experiment 3 and following the procedure described by Bryszak et al. (2019). Briefly, two infrared CH_4_ analyzers located in the feeding station were applied to measure CH_4_ concentration with a detection range of 0–500 ppm (0–625 mg/m^3^) for ambient air and 0–1000 ppm (0–1250 mg/m^3^) for feeding station (concentrates supplied site). Air samples (15 L/min) were constantly collected from the feed bins in the feeder stations through an 8-mm diameter polyethylene tube (length: 30 m) connected to a gas panel. The gas samples were distributed to the inlet port of the analyzer with a flow rate of 0.6 L/min. The infrared CH_4_ analyzers (Servomex 4000 Series, Servomex Ltd., Jarvis Brook, UK) were set up in a feeder station with an applied 1210 Gfx modules approach within the feeding time. The gas filter correlation technique was involved. Standard calibration gas (Multax, Zielonki-Parcela, Poland) containing 1210 ppm of CH_4_ in nitrogen gas (99.99%) was used to calibrate the analyzers. CH_4_ measurement data were stored on a computer using software with a database system (RS 232; AnaGaz, Wroclaw, Poland) after the experiment and measured at 3-s intervals.

#### 2.2.4 Gene expression analysis and microbial quantification and analysis

Based on the relative transcript abundance measured by real-time polymerase chain reaction (PCR), we measured six-gene expression [acetyl-CoA carboxylase 1 (*ACACA*), fatty acid synthase (*FASN*), lipoprotein lipase (*LPL*), stearoyl-CoA desaturase (*SCD*), fatty acid desaturase 1 (*FADS1*), and fatty acid elongase 5 (*ELOVL*5) in milk somatic cells (experiment 4), following the procedure described by Bryszak et al. (2020). The ruminal samples (experiments 1, 2, and 3) for quantification of protozoa (*Isotrichidae* and *Ophyroscolecidae*), total bacteria, and methanogens were analyzed using the protocols described by Szczechowiak et al. (2016). For the quantification of rumen bacteria (experiment 3), total deoxyribonucleic acid (DNA) was extracted from the rumen fluid using a QIAamp DNA Stool mini kit (Qiagen GmbH, Hilden, Germany). The quantitative analysis of particular bacteria was performed using a known starting concentration of bacterial DNA (25 ng/μl) on a QuantStudio 12 Flex PCR system (Life Technologies, Thermo Fisher Scientific, Waltham, MA, USA), following Bryszak et al. (2020). After the PCR run, the product size verification was also included by gel electrophoresis of samples. Purified genomic DNA from control strains was diluted to construct species-specific calibration curves (Szczechowiak et al., 2016). The calibration curves were then used to calculate species-specific DNA concentration using a specific number of DNA copies of total DNA preparations. The relative level of the DNA copy for each bacterium to the total bacteria species was calculated using the formula 2^−ΔCt^.

#### 2.2.5 Chemical analysis of milk

An infrared analyzer (Milko-Scan 255 A/S N; Foss Electric, Hillerød, Denmark) and a CombiFoss 6000 analyzer (Foss Electric, Hillerød, Denmark) were used to analyze basic milk parameters and urea concentration, respectively.

#### 2.2.6 Calculations

(1) The yield of energy-corrected milk (ECM) in experiment 4 was calculated according to Sjaunja et al. (1991): ECM = milk yield × (38.3 × fat, g/kg + 24.2 × protein, g/kg + 783.2)/3140.

(2) The in-situ degradation kinetics of DM, OM, and CP (experiment 5) were calculated according to the nonlinear model used by Ørskov and Mcdonald (1979), and the exponential model was used to determine the degradation of the nutrient at time t (D*t*) as follows:

D*t* = a + b × (1 - e ^-ct^)

where a is the soluble fraction (fraction washed out at t = 0, obtained from bags incubated for 0 h and corrected for particle loss); b is the insoluble degradable fraction; c is the fractional degradation rate (per hour), and t is the time in hours. The effective degradability (ED) was calculated as follows:

ED = *a* + (*b* × *c*/(*c* + k))

Following Chaves et al. (2006), the ruminal fractional flow rate (k) of 0.06/h was used.

### 2.3 Statistical analysis

All the data were analyzed using SAS statistical software and checked for normality using the UNIVARIATE procedure of SAS (Univ. Edition, version 9.4) and conformed to the normal distribution. The chemical composition and fatty acid profile of RC (n = 3) and FRC (n = 3) and between TMR (CONRC and FRC100) were tested with an independent *t*-test, where the means of both groups were compared using the PROC TEST procedure. In experiment 1, an independent *t*-test (PROC TTEST procedure) was used to compare the differences between treatments.

In experiment 2, all the data were analyzed using a one-way ANOVA model with PROC GLM procedure. The levels of the FRC used in the diet were considered a fixed factor, and each run was considered a random one. Linear, quadratic, and cubic contrasts were used to determine the effects of the FRC dose. In experiment 3, all the data were analyzed using the PROC MIXED procedure for a crossover design with the model containing group (dietary treatment sequence), period, and treatment as main effects, sampling time as repeated measures, and cow as a random effect. In experiment 4, the results were tested with an independent *t*-test where the means of both groups were compared through the PROC TTEST procedure. In experiment 5, the data on nutrient degradation were fitted with a nonlinear regression model. The NLIN procedure in SAS was used to calculate the parameter kinetics of *a*, *b* and *c*, and the data were then analyzed using the PROC TTEST procedure. In all experiments, the significance level was accepted at P < 0.05 and tended to be significant at 0.05 < P ≤ 0.10. All values from tables are shown as means with pooled standard error of means.

## 3 Results

### 3.1 Feed ingredients, chemical composition, cost of the daily diet, and fatty acid profile

The content of CP (P = 0.07) and neutral detergent fiber (P = 0.06) increased by 7% and 9.3%, respectively compared to the rapeseed cake. Regarding fatty acid content, FRC contained more C14:0 (P = 0.08), C16:0 (P = 0.07), C18:1, *cis*-9 (P = 0.08) than RC. Conversely, relatively low levels of C18:1 *cis*-11 (P = 0.07), C18:2 *cis*-9 *cis*-12 (P = 0.07), C18:3 *cis*-9 *cis*-12 *cis*-15 (P = 0.02) were found in FRC compared with the RC. Therefore, the sum of monounsaturated fatty acids (MUFA; P = 0.07) tended to increase and the sum of polyunsaturated fatty acids (PUFA; P = 0.05), n-6 FA (P = 0.08) and n-3 FA (P = 0.02) decreased in FRC. In the case of TMR, FRC100 contained lesser C18:2 *cis*-9 *cis*-12 (P = 0.08) than the CONRC diet and tended to decrease the sum of PUFA (P = 0.06) and n-6 FA (P = 0.08).

### 3.2 Ruminal fermentation characteristics, microbial counts, and fatty acid profile (Experiment 1)

**Fig. 1:**
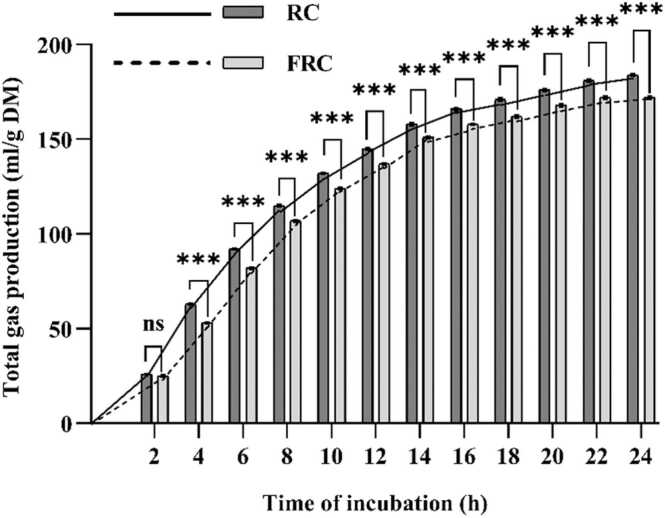
Gas production from fermented rapeseed cake (FRC) or rapeseed cake (RC) incubated with rumen fluid in the in vitro Hohenheim gas production test. Data presented as the real-time measurement of total gas produced as mL/g of RC or FRC (DM basis). Total gas production was recorded throughout a 24-h incubation. Five replicates of each treatment were used in 3 runs (n = 15). RC = Diet with rapeseed cake as the sole substrate. FRC = Diet with fermented rapeseed cake as the sole substrate. ns represents no significant change compared with RC; ***significant differ at P < 0.01. Error bars represent SEM.

Ruminal pH, ammonia concentration, IVDMD, and IVOMD increased in the FRC group (P < 0.01; Table 2). Decreased CH_4_ production (per gram of DM, total gas, IVDMD, or IVOMD) (P < 0.01) was observed with the FRC diet. The molar proportion of acetate, isobutyrate, and isovalerate decreased in the FRC group (P ≤ 0.01). However, the molar proportion of propionate was higher, and the A/P ratio was lower for FRC (P < 0.01) than RC. Significant (P ≤ 0.01) decreases in archaea, and *Isotrichidae* populations were observed in the experimental group. The higher numbers of bacteria, total protozoa, and *Ophyroscolecidae* were noted for FRC (P < 0.01). Total gas production from the FRC was lower (P < 0.01) by 6.3% than the RC after 24 h incubation (Fig. 1). FRC decreased C18:0 (P = 0.04) content compared with the RC (Table 3). However, C16:0 concentration (P < 0.01) increased with FRC. C18:1 *trans-*11 (P = 0.08) content tended to increase with FRC, while C18:1 *cis-*9 (P = 0.06) and n-6 FA (P = 0.08) content tended to decrease with FRC.

**Table 2:** Effects of fermented rapeseed cake (FRC) on ruminal fermentation characteristics and microbial counts in a Hohenheim glass syringes system (experiment 1; n = 15).

Parameters	RC	FRC	SEM	P-value
pH	6.17	6.21	0.005	< 0.01
NH_3,_ m*M*	19.8	23.3	0.51	< 0.01
IVDMD, g/kg	524	569	6.81	< 0.01
IVOMD, g/kg	577	619	8.38	< 0.01
CH_4_, mmole	0.67	0.45	0.03	< 0.01
Total gas, mL/g DM	183	171	1.45	< 0.01
CH_4_, mmole/g DM	3.35	2.23	0.17	< 0.01
CH_4_/total gas, mmole/L	18.1	13.0	0.87	< 0.01
CH_4_/IVDMD, mmole/g	5.95	3.55	0.34	< 0.01
CH_4_/IVOMD, mmole/g	6.53	4.63	0.32	< 0.01
Total VFA, m*M*	77.6	78.1	0.56	0.66
VFA proportion, mol/100 mol				
Acetate (A)	58.5	56.6	0.20	< 0.01
Propionate (P)	26.0	28.3	0.24	< 0.01
Isobutyrate	1.37	1.20	0.03	< 0.01
Butyrate	9.02	8.80	0.07	0.14
Isovalerate	2.04	1.82	0.04	0.01
Valerate	3.07	3.27	0.06	0.10
A/P ratio	2.24	2.00	0.03	< 0.01
Total bacteria, × 10^8^ /mL	2.62	3.79	0.12	< 0.01
Archaea, × 10^6^/mL	3.95	3.35	0.12	0.01
Total protozoa, × 10^4^/mL	0.98	1.43	0.05	< 0.01
*Isotrichidae*, × 10^4^/mL	0.02	0.008	0.002	< 0.01
*Ophyroscolecidae*, × 10^4^/mL	0.96	1.42	0.06	< 0.01

RC: sole raw rapeseed cake as substrate; FRC: sole fermented rapeseed cake as substrate; NH3: ammonia; IVDMD: in vitro dry matter degradability; IVOMD: in vitro organic matter degradability; CH4: methane; DM: dry matter; VFA; volatile fatty acids; SEM: standard error of means. Treatments are considered significantly different at P < 0.05.

**Table 3:** The effect of fermented rapeseed cake (FRC) supplementation on ruminal fatty acid (FA) concentrations (g/100 g of FA) in a Hohenheim glass syringes system (experiment 1; n = 15).

Item	RC	FRC	SEM	P-value
*Saturated fatty acids*				
C12:0	1.05	1.18	0.104	0.54
C14:0	1.23	1.41	0.06	0.11
C15:0	0.81	0.92	0.03	0.12
C16:0	14.7	16.4	0.32	< 0.01
C18:0	32.4	29.6	0.63	0.04
*Monounsaturated fatty acids*				
C18:1 *trans-*10	2.51	2.30	0.07	0.10
C18:1 *trans-*11	10.5	11.7	0.36	0.08
C18:1 *cis-*9	16.8	14.7	0.57	0.06
*Polyunsaturated fatty acids*				
C18:2 *cis*-9, *cis*-12	2.30	1.87	0.14	0.13
C18:2 *cis*-9, *trans*-11	0.61	0.63	0.012	0.40
C18:2 *trans*-10, *cis*-12	0.06	0.05	0.006	0.84
C18:3 *cis*-9, *cis*-12, *cis*-15	0.29	0.31	0.014	0.49
Sum of other FA^1^	16.6	17.4	0.43	0.43
Sum of SFA	54.6	54.8	0.56	0.84
Sum of UFA	45.4	45.2	0.56	0.84
Sum of MUFA	41.5	41.8	0.59	0.80
Sum of PUFA	3.95	3.38	0.20	0.16
Sum of n-6 FA	2.67	2.17	0.14	0.08
Sum of n-3 FA	0.58	0.54	0.02	0.46
n-6/n-3 FA ratio	4.18	4.21	0.25	0.95

RC: sole raw rapeseed cake as substrate; FRC: sole fermented rapeseed cake as substrate; SFA: saturated fatty acids; UFA: unsaturated fatty acids; MUFA: monounsaturated fatty acids; PUFA: polyunsaturated fatty acids; SEM: standard error of means. Treatments are considered significantly different at P < 0.05.

^1^Other FA include C6, C8:0, C10:0, C11:0, C17:0, C10:1, C14:1, C15:1, C16:1, C17:1, C12 *iso*, C12 *anteiso*, C13:0, C14 *anteiso*, C15 *anteiso*, C16 *anteiso*, C16*iso*, C16:1 *trans*, C17 *iso*, C17 *anteiso*, C18:1 *trans*-5, C18:1 *trans*-6–8, C18:1 *trans*-9, C18:1 *cis*-11, C18:1 *cis*-12, C18:1 *cis*-13, C18:1 *cis*-14, C19:0, C18:2 *cis*-9,*cis*-15, C18:3 *cis*-9*,trans*-11*,cis*-15, C20:1 *trans*, C18:3 n − 6, C20:5n-3, C22:5n-3, C22:6n-3, C21:0, C22:0, C23:0, C24:0, and C24:1.

### 3.3 Ruminal fluid characteristics, microbial counts, and fatty acid profile in relation to the FRC dose (Experiment 2)

Ruminal ammonia concentration, IVDMD, and IVOMD increased linearly (P < 0.01) with increasing concentrations of FRC (Table 4). The greatest value of these three parameters was observed when FRC fully replaced RC. There was a linear (P ≤ 0.01) and cubic (P ≤ 0.02) reduction in CH_4_ and total gas production as the doses of FRC increased. The molar proportions of acetate and butyrate decreased linearly (P < 0.01) with increasing doses of FRC. Nonetheless, the molar proportion of propionate, isobutyrate, isovalerate, and total VFA increased linearly (P < 0.01) in the experimental groups and also exhibited cubic (P ≤ 0.03) effects. In addition, the A/P ratio decreased linearly (P < 0.01) and cubically (P < 0.01) with increasing FRC in the TMR. The inclusion of FRC in the diet linearly reduced the archaeal population (P < 0.01). The total bacterial population increased linearly (P < 0.01) and cubically (P = 0.04) with increasing amounts of FRC. Supplementation with FRC caused a linear (P < 0.01) and quadratic (P = 0.04) increase in the population of total protozoa and *Ophyroscolecidae* and a linear and quadratic decrease in the population of *Isotrichidae* (P < 0.01), with a tendency to decrease cubically (P = 0.09). All FA except the concentration of C12:0 showed a significant difference with a dose-response (Table 5). C14:0, C15:0, and C16:0 FA content changed in a linear dose-dependent manner (P ≤ 0.04). The FRC diet reduced the concentration of C18:0 in ruminal fluid quadratically and cubically (P ≤ 0.01), with the lowest content at 28.75 g/kg of FRC. The concentrations of C18:1 *trans*-10 (lowest at 28.75 and 57.5 g/kg), C18:1 *trans-*11 (lowest at 28.75), and C18:1 *cis-*9 (lowest at CONRC) altered quadratically. The content of C18:2 *cis*-9 *cis*-12 (quadratic effect with highest at 57.5 g/kg), C18:2 *cis*-9 *trans*-11 (quadratic effect with lowest at 57.5 g/kg), C18:2 *trans*-10 *cis*-12 (quadratic and cubic effect with lowest at 86.25 g/kg), and C18:3 *cis*-9 *cis*-12 *cis*-15 (linear and quadratic effect) changed in a dose-dependent manner. Apart from that, unsaturated fatty acids (UFA) and MUFA increased quadratically (P < 0.01) depending upon the dose, with the highest at 28.75 g/kg of FRC. PUFA increased cubically (P = 0.02), n-6 FA increased linearly, and n-3 FA decreased quadratically with the lowest value at 86.25 g/kg.

**Table 4:** Effects of fermented rapeseed cake (FRC) on ruminal fermentation characteristics and microbial counts in a batch culture system (experiment 2; n = 15).

Item	CONRC	FRC				SEM	Contrast		
		28.75	57.5	86.25	115		Linear	Quadratic	Cubic
pH	6.23	6.26	6.23	6.24	6.26	0.01	0.25	0.43	0.10
NH_3,_ m*M*	15.1	16.2	16.1	16.6	17.7	0.23	< 0.01	0.73	0.24
IVDMD, g/kg	597	596	595	601	619	2.1	< 0.01	< 0.01	0.32
IVOMD, g/kg	619	620	622	626	639	2.1	< 0.01	0.13	0.54
CH_4_, mmole	1.18	1.12	1.13	1.12	1.06	0.01	< 0.01	0.69	0.02
Total gas, mL/g DM	371	364	365	363	353	1.17	< 0.01	0.19	0.01
CH_4_, mmole/g DM	2.95	2.80	2.83	2.80	2.64	0.02	< 0.01	0.46	0.02
CH_4_/total gas, mmole/L	7.96	7.75	7.75	7.74	7.48	0.03	< 0.01	0.63	< 0.01
CH_4_/IVDMD, mmole/g	4.96	4.72	4.79	4.69	4.27	0.04	< 0.01	0.04	< 0.01
CH_4_/IVOMD, mmole/g	5.13	4.75	4.89	4.80	4.38	0.04	< 0.01	0.30	< 0.01
Total VFA, m*M*	77.7	82.7	82.1	83.0	86.8	0.58	< 0.01	0.79	0.02
VFA proportion, mol/100 mol									
Acetate (A)	59.5	58.9	56.3	56.6	54.2	0.35	< 0.01	0.73	0.98
Propionate (P)	21.9	21.2	25.5	25.4	26.4	0.36	< 0.01	0.62	0.03
Isobutyrate	1.25	1.13	1.29	2.09	2.47	0.07	< 0.01	< 0.01	< 0.01
Butyrate	13.4	13.1	12.6	12.5	12.3	0.13	< 0.01	0.44	0.85
Isovalerate	1.58	1.91	1.60	1.92	2.15	0.04	< 0.01	0.02	< 0.01
Valerate	2.22	2.19	1.82	2.31	2.29	0.03	0.13	< 0.01	0.32
A/P ratio	2.71	2.88	2.27	2.22	2.09	0.05	< 0.01	0.95	< 0.01
Total bacteria, × 10^8^ /mL	4.47	4.50	4.49	5.53	5.31	0.11	< 0.01	0.40	0.04
Archaea, × 10^6^/mL	6.14	5.21	3.95	3.28	2.34	0.27	< 0.01	0.54	0.95
Total protozoa, × 10^4^/mL	1.72	2.42	3.10	3.37	3.88	0.10	< 0.01	0.04	0.41
*Isotrichidae*, × 10^4^/mL	0.03	0.03	0.02	0.02	0.01	0.001	< 0.01	< 0.01	0.09
*Ophyroscolecidae*, × 10^4^/mL	1.69	2.39	3.09	3.35	3.87	0.10	< 0.01	0.04	0.41

CONRC: a control group fed a total mixed ration (TMR), which included 115 g/kg diet of raw rapeseed cake (DM basis); FRC: the level of fermented rapeseed cake used in the incubated substrate replaced raw rapeseed cake in the control; SEM: standard error of means; Contrast: polynomial contrast results due to the level of FRC used in the diet; NH3: ammonia; IVDMD: in vitro dry matter degradability; IVOMD: in vitro organic matter degradability; CH4: methane; DM: dry matter; VFA; volatile fatty acids.

**Table 5:** The effect of fermented rapeseed cake (FRC) supplementation on ruminal fatty acid (FA) concentrations (g/100 g of FA) in a batch culture system (experiment 2; n = 15).

Item	CONRC	FRC				SEM	Contrast		
		28.75	57.5	86.25	115		Linear	Quadratic	Cubic
*Saturated fatty acids*									
C12:0	1.07	1.28	1.09	1.05	1.20	0.06	0.96	0.84	0.12
C14:0	1.88	1.83	1.92	2.15	2.10	0.04	< 0.01	0.67	0.11
C15:0	1.86	1.65	1.75	2.01	1.90	0.03	0.04	0.12	< 0.01
C16:0	22.1	22.9	24.2	23.4	23.4	0.26	0.04	0.05	0.90
C18:0	42.8	37.1	38.8	38.8	39.4	0.47	0.07	< 0.01	0.01
*Monounsaturated fatty acids*									
C18:1 *trans-*10	0.89	0.75	0.75	0.82	0.91	0.02	0.29	< 0.01	0.32
C18:1 *trans-*11	4.66	3.92	4.16	4.28	4.29	0.07	0.37	< 0.01	0.01
C18:1 *cis-*9	7.49	11.7	9.43	8.68	8.11	0.31	0.31	< 0.01	< 0.01
*Polyunsaturated fatty acids*									
C18:2 *cis*-9, *cis*-12	2.33	3.37	3.39	3.13	3.11	0.12	0.08	< 0.01	0.06
C18:2 *cis*-9, *trans*-11	0.80	0.73	0.72	0.74	0.77	0.01	0.32	< 0.01	0.36
C18:2 *trans*-10, *cis*-12	0.14	0.17	0.08	0.07	0.10	0.01	< 0.01	0.05	< 0.01
C18:3 *cis*-9, *cis*-12, *cis*-15	0.56	0.54	0.59	0.63	0.79	0.02	< 0.01	0.01	0.88
Sum of other FA^1^	14.7	13.1	14.3	14.1	13.9	0.37	0.87	0.12	0.35
Sum of SFA	75.6	72.3	73.3	74.0	73.9	0.27	0.35	< 0.01	< 0.01
Sum of UFA	24.4	27.7	26.7	26.0	26.1	0.27	0.35	< 0.01	< 0.01
Sum of MUFA	19.2	21.7	20.8	20.5	20.2	0.22	0.58	< 0.01	0.05
Sum of PUFA	5.23	6.01	5.96	5.54	5.9	0.11	0.28	0.09	0.02
Sum of n-6 FA	2.78	3.83	3.80	3.59	3.57	0.12	0.07	< 0.01	0.05
Sum of n-3 FA	1.69	1.45	1.46	1.35	1.49	0.04	0.04	0.03	0.97
n-6/n-3 FA ratio	1.66	3.17	2.27	3.05	2.48	0.15	0.11	0.03	0.26

CONRC: a control group fed a total mixed ration (TMR), which included 115 g/kg diet of raw rapeseed cake (DM basis); FRC: the level of fermented rapeseed cake used in the incubated substrate replaced raw rapeseed cake in the control; SFA: saturated fatty acids; UFA: unsaturated fatty acids; MUFA: monounsaturated fatty acids; PUFA: polyunsaturated fatty acids; SEM: standard error of means.

^1^Other FA include C6, C8:0, C10:0, C11:0, C17:0, C10:1, C14:1, C15:1, C16:1, C17:1, C12 *iso*, C12 *anteiso*, C13:0, C14 *anteiso*, C15 *anteiso*, C16 *anteiso*, C16 *iso*, C16:1 *trans*, C17 *iso*, C17 *anteiso*, C18:1 *trans*-5, C18:1 *trans*-6–8, C18:1 *trans*-9, C18:1 *cis*-11, C18:1 *cis*-12, C18:1 *cis*-13, C18:1 *cis*-14, C19:0, C18:2 *cis*-9,*cis*-15, C18:3 *cis*-9*,trans*-11*,cis*-15, C20:1 *trans*, C18:3 n − 6, C20:5n-3, C22:5n-3, C22:6n-3, C21:0, C22:0, C23:0, C24:0, and C24:1.

### 3.4 Ruminal fluid parameters, microbial counts, and fatty acid profile (Experiment 3)

The ruminal pH in the cannulated cows fed the FRC100 diet was lower (P < 0.01) than in the CONRC group (Table 6). In contrast, ammonia concentration was higher (P < 0.01), and these variables depended on the time of feeding (P < 0.01). When FRC100 treatment was used, the molar proportion of propionate and valerate increased, whereas acetate, butyrate, isovalerate, and A/P ratio decreased (P ≤ 0.01). Time-dependent variation was also observed in all the individual VFA proportions and total VFA (P ≤ 0.01). The microbial counts of total protozoa, *Isotrichidae*, and *Ophyroscolecidae* differed within the group (P < 0.01), but they did not show a time-dependent effect, except for *Isotrichidae* (P < 0.01). Most FA concentrations differ between treatments and time points (Table 7). The application of FRC100 caused a decrease in the proportions of saturated fatty acids (SFA) such as C14:0, C15:0, C16:0, C18:0, and the sum of SFA (P < 0.01) except for an increase in the proportion of C12:0 in the experimental groups (P < 0.01). Compared to CONRC, FRC100 altered C18:2 conjugated isomers, and the differences were time-dependent (P < 0.01).

**Table 6:** Effect of feeding fermented rapeseed cake (FRC) to cannulated dairy cows on ruminal fermentation characteristics and microbial counts (experiment 3; n = 4).

Item^a^	0 h^b^		3 h^c^		6 h^d^		Group^e^		SEM^f^	P-value^g^		
	CONRC	FRC100	CONRC	FRC100	CONRC	FRC100	CONRC	FRC100		G	H	GxH
pH	6.07	5.98	6.19	5.95	6.23	6.19	6.16	6.04	0.02	< 0.01	< 0.01	0.06
NH_3_, m*M*	5.68	6.14	6.57	8.13	6.11	7.54	6.12	7.33	0.15	< 0.01	< 0.01	0.09
Total VFA, m*M*	113	110	124	128	126	138	121	125	1.49	0.09	< 0.01	0.06
VFA proportion, mol/100 mol												
Acetate (A)	64.1	60.9	60.6	57.5	62.1	58.1	62.3	58.8	0.28	< 0.01	< 0.01	0.47
Propionate (P)	19.8	24.5	20.8	25.2	21.1	26.3	20.6	25.3	0.30	< 0.01	< 0.01	0.57
Isobutyrate	1.08	1.23	1.05	1.09	0.90	0.99	1.01	1.10	0.04	0.09	0.01	0.73
Butyrate	12.1	11.7	13.6	12.7	12.5	11.9	12.7	12.1	0.104	< 0.01	< 0.01	0.41
Isovalerate	1.63	0.86	2.01	1.09	1.74	0.81	1.79	0.92	0.05	< 0.01	< 0.01	0.13
Valerate	1.46	1.71	1.92	2.11	1.68	1.78	1.69	1.87	0.03	0.01	< 0.01	0.47
A/P	3.24	2.46	2.92	2.34	2.95	2.25	3.03	2.35	0.04	< 0.01	< 0.01	0.28
Microbial population												
Total protozoa, × 10^d^/mL	15.8	9.52	19.7	10.6	16.6	9.28	17.4	9.80	0.64	< 0.01	0.11	0.55
*Isotrichidae*, × 10^d^/mL	0.08	0.04	0.49	0.29	0.26	0.22	0.28	0.19	0.02	< 0.01	< 0.01	< 0.01
*Ophyroscolecidae*, × 10^d^/mL	15.7	9.50	19.2	10.3	16.4	9.00	17.1	9.60	0.60	< 0.01	0.17	0.58

^a,b,c^Means of different groups in the same row indicate significant differences at P < 0.05.

a NH_3_: ammonia; VFA: volatile fatty acids.

b The ruminal fluid was obtained from each cannulated cow from three locations in the midventral sac of the rumen before morning feeding (0 h).

c The ruminal fluid was obtained from each cannulated cow from three locations in the midventral sac of the rumen 3 h after morning feeding.

d The ruminal fluid was obtained from each cannulated cow from three locations in the midventral sac of the rumen 6 h after morning feeding.

e CONRC: a control group fed a total mixed ration (TMR), which included 115 g/kg diet of raw rapeseed cake (DM basis); FRC100: TMR which included 115 g/kg diet of fermented rapeseed cake (DM basis) instead of raw rapeseed cake.

f SEM: standard error of means.

g Probability of effects: G = effects of group, CONRC vs. FRC100 diets; H = effect of digest time in different time points; G × H = interaction between the group and digest time.

**Table 7:** Fatty acid (FA) concentration (g/100 g of FA) in the ruminal fluid of cannulated dairy cows fed a diet with fermented rapeseed cake (FRC) (experiment 3; n = 4).

Item^a^	0 h		3 h		6 h		Group^b^		SEM^c^	P-value^d^		
	CONRC	FRC100	CONRC	FRC100	CONRC	FRC100	CONRC	FRC100		G	H	G×H
*Saturated fatty acids*												
C12:0	0.28	0.38	0.26	0.30	0.28	0.30	0.27	0.33	0.007	< 0.01	< 0.01	0.04
C14:0	0.11	0.08	0.12	0.09	0.13	0.12	0.12	0.10	0.003	< 0.01	< 0.01	0.04
C15:0	1.19	1.05	1.18	0.95	1.16	1.02	1.18	1.00	0.011	< 0.01	< 0.01	< 0.01
C16:0	19.0	17.2	19.3	16.4	19.4	16.3	19.2	16.6	0.15	< 0.01	0.10	< 0.01
C18:0	51.8	48.8	50.1	47.2	49.8	51.4	50.5	48.9	0.29	< 0.01	< 0.01	< 0.01
*Monounsaturated fatty acids*												
C18:1 *trans-*10	2.25	2.89	2.21	2.98	2.04	2.80	2.18	2.90	0.06	< 0.01	0.25	0.77
C18:1 *trans-*11	1.94	1.95	2.41	2.40	2.35	2.34	2.26	2.23	0.04	0.83	< 0.01	0.98
C18:1 *cis-*9	7.01	9.31	7.26	10.6	7.81	8.92	7.36	9.73	0.17	< 0.01	< 0.01	< 0.01
*Polyunsaturated fatty acids*												
C18:2 *cis*-9, *cis*-12	2.97	3.95	3.99	4.38	3.86	3.31	3.64	3.95	0.12	0.19	0.02	0.02
C18:2 *cis*-9, *trans*-11	0.68	0.50	0.46	0.66	0.79	0.54	0.63	0.58	0.04	0.22	0.09	< 0.01
C18:2 *trans*-10, *cis*-12	0.29	0.17	0.27	0.22	0.20	0.15	0.26	0.19	0.009	< 0.01	< 0.01	0.03
C18:3 *cis*-9, *cis*-12, *cis*-15	0.75	0.93	0.72	0.94	0.76	0.77	0.74	0.89	0.02	< 0.01	0.04	0.01
Sum of other FA^e^	11.6	12.2	11.4	14.5	11.5	11.6	11.5	12.9	0.38	0.10	0.29	0.22
Sum of SFA	76.6	71.5	75.8	68.8	75.4	73.2	75.9	70.8	0.37	< 0.01	< 0.01	< 0.01
Sum of UFA	23.4	28.5	24.2	31.2	24.6	26.8	24.1	29.2	0.37	< 0.01	< 0.01	< 0.01
Sum of MUFA	18.3	22.3	18.3	24.3	18.7	21.7	18.4	22.9	0.31	< 0.01	< 0.01	< 0.01
Sum of PUFA	5.16	6.28	5.92	6.90	5.93	5.16	5.70	6.24	0.12	0.04	< 0.01	< 0.01
Sum of n-6 FA	3.89	5.22	4.96	5.67	4.76	4.29	4.55	5.15	0.13	0.03	0.01	< 0.01
Sum of n-3 FA	0.88	1.05	0.83	1.04	0.88	0.87	0.86	1.00	0.02	< 0.01	0.07	0.02
n-6/n-3 FA ratio	4.45	5.04	5.43	5.33	5.00	4.85	5.01	5.09	0.10	0.57	0.03	0.24

^a,b,c,d^Means of different groups in the same row indicate significant differences at P < 0.05.

a SFA: saturated fatty acids; UFA: unsaturated fatty acids; MUFA: monounsaturated fatty acids; PUFA: polyunsaturated fatty acids.

b CONRC: a control group fed a total mixed ration (TMR), which included 115 g/kg diet of raw rapeseed cake (DM basis); FRC100: TMR, which included 115 g/kg diet of fermented rapeseed cake (DM basis) instead of raw rapeseed cake.

c SEM: standard error of means.

d Probability of effects: G = effects of group, CONRC vs. FRC100 diets; H = effect of digest time in different time points; G × H = interaction between the group and digest time.

e Other FA include C6, C8:0, C10:0, C11:0, C17:0, C10:1, C12 *iso*, C12 *anteiso*, C13:0, C14:1, C15:1, C16:1, C17:1, C14 *anteiso*, C15 *anteiso*, C16 *anteiso*, C16 *iso*, C16:1 *trans*, C17 *iso*, C17 *anteiso*, C18:1 *trans*-5, C18:1 *trans*-6–8, C18:1 *trans*-9, C18:1 *trans*-15, C18:1 *cis*-11, C18:1 *cis*-12, C18:1 *cis*-13, C18:1 *cis*-14, C19:0, C18:1 *cis*-15, C18:2 *cis*-9,*cis*-12, C18:2 *cis*-9,*cis*-15, C20:0, C20:1 *trans,* C18:3n-6, C21:0, C20:2, C22:0, C20:5n-3, C22:6n-3, C20:3 n − 6, C22:1n-9, C20:3 n − 3, C23:0, C22:2, C24:0, and C24:1.

Decreased (P ≤ 0.04) CH_4_ production (g/d) was noted in FRC100 compared with the CONRC. The populations of *Ruminococcus flavefaciens*, *Streptococcus bovis*, *Butyrivibrio proteoclasticus*, *Lactobacillus* spp., and *Megasphaera elsdenii* were significantly higher in the FRC100 than in the CONRC group (P ≤ 0.04). In contrast, *Ruminococcus albus*, *Butyrivibrio fibrisolvens*, and archaea counts were lesser (P < 0.01) in the FRC100 group (Table 8).

**Table 8:** The effect of fermented rapeseed cake (FRC) supplementation on in vivo ruminal microorganisms, methane emission, and total-tract digestibility (experiment 3; n = 4).

Item^a^	CONRC	FRC100	SEM	P-value
Microbial population				
Total bacteria × 10^9^ /mL	7.46	7.30	0.20	0.69
Archaea, × 10^8^/ mL	6.88	4.56	0.23	< 0.01
Ruminococcus flavefaciens*	1.00	2.07	0.26	0.04
Fibrobacter succinogenes*	1.00	0.82	0.21	0.66
Streptococcus bovis*	1.00	3.03	0.39	< 0.01
*Prevotella* spp.*	1.00	1.23	0.11	0.30
Butyrivibrio proteoclasticus*	1.00	1.55	0.13	0.03
Ruminococcus albus*	1.00	0.59	0.08	< 0.01
Butyrivibrio fibrisolvens*	1.00	0.53	0.08	< 0.01
*Lactobacillus* spp.*	1.00	4.86	0.72	< 0.01
Megasphaera elsdenii*	1.00	2.06	0.24	0.02
DMI	23.1	23.6	0.17	0.17
Total-tract digestibility, g/kg				
DM	581	583	6.28	0.89
OM	601	608	8.15	0.66
CP	534	564	12.9	0.24
NDF	435	470	7.34	0.04
CH_4_, g/d	413	354	14.5	0.04
CO_2,_ g/d	11,317	10,827	419	0.57

CONRC: a control group fed a total mixed ration (TMR), which included 115 g/kg diet of raw rapeseed cake (DM basis); FRC100: TMR which included 115 g/kg diet of fermented rapeseed cake (DM basis) instead of raw rapeseed cake.

SEM: standard error of means.

* Expressed as an arbitrary unit relative to the total bacterial gene copy abundance of the control

a DMI: dry matter intake; DM: dry matter; OM: organic matter; CP: crude protein; CH_4_: methane.

### 3.5 Milk production and composition in dairy cows (Experiment 4)

**Fig. 2:**
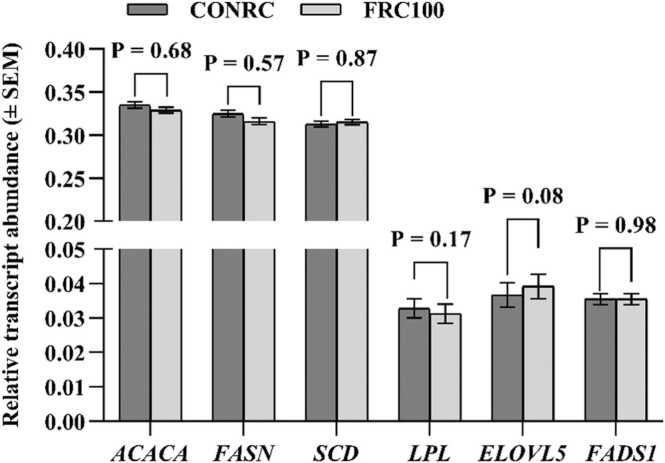
The effect of fermented rapeseed cake (FRC; n = 10) supplementation on the expression of six genes [*acetyl-CoA carboxylase 1 (ACACA), fatty acid synthase (FASN), stearoyl-CoA desaturase (SCD), lipoprotein lipase (LPL), fatty acid elongase 5 (ELOVL5)*, and *fatty acid desaturase 1 (FADS1)*] in the somatic cells of milk of lactating cows. Error bars represent SEM.

Milk production and its component yields were similar in both groups except fat yield, which tended to decrease (P = 0.06) due to feeding of FRC (Table 9). The DMI presented in the table is the sum of intake by the feeder station individually recorded daily and the PMR intake registered daily for each group separately. Among the milk composition, the concentration of protein tended to increase (P = 0.09), fat tended to decrease (P = 0.09), and urea increased (P = 0.04) in the FRC100 group. CH_4_ concentration was significantly lower in FRC100 than in CONRC (P = 0.01). Milk FA had higher concentrations of C18:2 *cis*-9 *trans*-11, C18:*2 trans*-10 *cis*-12, and MUFA (P ≤ 0.04), and lower concentration of C15:0 and n-6/n-3 FA ratio (P ≤ 0.04) in cows fed FRC100 (Table 10). There were no significant differences between treatments in desaturase, thrombogenic, and atherogenic indices (Table 10). The application of FRC100 tended to increase (P = 0.08) the mRNA abundance of the ELOVL5 gene, whereas the abundance of the other genes did not differ (Fig. 2).

**Table 9:** Effect of feeding fermented rapeseed cake (FRC) to dairy cows on the milk production, yield, and composition (experiment 4; n = 10).

Parameters	CONRC	FRC100	SEM	P-value
DMI	23.1	23.3	0.65	0.39
Milk yield, kg/d	36.3	36.0	0.53	0.77
Milk composition				
Protein, g/kg	32.4	32.9	0.16	0.09
Fat, g/kg	38.9	34.9	1.12	0.09
Lactose, g/kg	46.6	46.8	0.23	0.57
Casein, g/kg	28.3	28.2	0.59	0.94
TS, g/kg	118	115	1.16	0.37
SNF, g/kg	79.3	79.7	0.36	0.64
Energy, MJ/kg	2.91	2.75	0.05	0.11
Urea, mg/L	223	252	7.30	0.04
Yield				
Protein, g/d	1171	1181	15.4	0.74
Fat, g/d	1411	1254	37.8	0.06
Lactose, g/d	1687	1683	24.1	0.94
Casein, g/d	1035	1021	19.0	0.71
ECM, kg/d	34.1	33.9	0.43	0.77
Energy, MJ/d	105	99.4	1.72	0.13
Methane, ppm	207	184	3.77	0.01

CONRC: a control group fed a total mixed ration (TMR), which included 115 g/kg diet of raw rapeseed cake (DM basis); FRC100: TMR which included 115 g/kg diet of fermented rapeseed cake (DM basis) instead of raw rapeseed cake; DMI: dry matter intake; TS: total solids; SNF: solid non-fat; ECM: energy corrected milk; SEM: standard error of means. Treatments are considered significantly different at P < 0.05.

**Table 10:** Effect of feeding fermented rapeseed cake (FRC) to dairy cows on fatty acid (FA) concentrations (g/100 g of FA) in milk (experiment 4; n = 10).

Item	CONRC	FRC100	SEM	P-value
*Saturated fatty acids*				
C12:0	3.88	4.17	0.08	0.06
C14:0	11.9	12.5	0.15	0.08
C15:0	1.40	1.30	0.02	0.04
C16:0	30.4	29.1	0.38	0.06
C18:0	9.93	10.2	0.24	0.56
*Monounsaturated fatty acids*				
C18:1 *trans*-10	0.31	0.33	0.014	0.47
C18:1 *trans*-11	0.37	0.42	0.02	0.09
C18:1 *cis*-9	21.7	21.8	0.40	0.96
*Polyunsaturated fatty acids*				
C18:2 *cis*-9, *cis*-12	3.91	3.56	0.12	0.05
C18:2 *cis*-9, *trans*-11	0.50	0.57	0.02	< 0.01
C18:2 *trans*-10, *cis*-12	0.09	0.12	0.004	< 0.01
C18:3 *cis*-9, *cis*-12, *cis*-15	0.40	0.43	0.009	0.14
Other FA^a^	15.1	15.3	0.12	0.58
Sum of SFA	66.9	65.3	0.38	0.05
Sum of UFA	33.1	34.7	0.38	0.05
Sum of MUFA	27.9	29.3	0.34	0.04
Sum of PUFA	5.21	5.35	0.12	0.57
Sum of n-6 FA	4.41	4.08	0.13	0.08
Sum of n-3 FA	0.51	0.55	0.01	0.07
n-6/n-3 FA ratio	8.80	7.63	0.28	< 0.01
DI^b^	0.31	0.31	0.005	0.61
DI C14:1^b^	0.10	0.09	0.002	0.18
DI C16:1^b^	0.05	0.04	0.001	0.08
DI C18:1^b^	0.69	0.68	0.006	0.64
DI RA/(VA+RA)^b^	0.56	0.57	0.02	0.75
Thrombogenic index^c^	2.87	2.74	0.05	0.19
Atherogenic index^d^	2.73	2.63	0.04	0.31

CONRC: a control group fed a total mixed ration (TMR), which included 115 g/kg diet of raw rapeseed cake (DM basis); FRC100: TMR which included 115 g/kg diet of fermented rapeseed cake (DM basis) instead of raw rapeseed cake; SFA: saturated fatty acids; UFA: unsaturated fatty acids; MUFA: monounsaturated fatty acids; PUFA: polyunsaturated fatty acids; SEM: standard error of means. Treatments are considered significantly different at P < 0.05.

a Other FA include C6, C8:0, C10:0, C11:0, C17:0, C10:1, C14:1, C15:1, C16:1, C17:1, C12 *iso*, C12 *anteiso*, C13:0, C14 *anteiso*, C15 *anteiso*, C16 *anteiso*, C16*iso*, C16:1 *trans*, C17 *iso*, C17 *anteiso*, C18:1 *trans*-5, C18:1 *trans*-6–8, C18:1 *trans*-9, C18:1 *cis*-11, C18:1 *cis*-12, C18:1 *cis*-13, C18:1 *cis*-14, C19:0, C18:2 *cis*-9,*cis*-15, C18:3 *cis*-9*,trans*-11*,cis*-15, C20:1 *trans*, C18:3 n − 6, C20:5n-3, C22:5n-3, C22:6n-3, C21:0, C22:0, C23:0, C24:0, and C24:1.

b According to Brogna et al. (2011). DI: desaturation index; VA: vaccenic acid.

c According to Ulbricht and Southgate (1991).

d According to Chilliard et al. (2003).

### 3.6 *In sacco* ruminal degradation kinetics, potential degradation, and effective degradation (Experiment 5)

The parameters of DM degradation kinetics differed significantly between the RC and the FRC, except for the fractional disappearance rate *c* (Table 11). The FRC contained a higher soluble fraction *a,* and a lower slowly degradable fraction *b* (P < 0.01). The potential and effective degradability of DM were higher in the FRC group than in the RC group (P < 0.01). The *a*, ED, and PD of OM degradation were higher for FRC (P < 0.01), whereas the slowly degradable fraction *b* was lower in the experimental group (P < 0.01). For CP degradation, FRC showed the same trend as in the case of OM parameters.

**Table 11:** *In sacco* ruminal degradation kinetics^a^, potential (PD)^b^ and effective (ED)^c^ degradation of raw rapeseed cake (RC) and fermented rapeseed cake (FRC) (experiment 5; n = 6).

Parameter	RC	FRC	SEM	P-value
Dry matter-degradation parameters				
a	0.24	0.37	0.019	< 0.01
b	0.57	0.47	0.016	< 0.01
c	0.20	0.20	0.005	0.61
PD	0.82	0.84	0.003	< 0.01
ED	0.69	0.73	0.007	< 0.01
Organic matter-degradation parameters				
a	0.24	0.34	0.015	< 0.01
b	0.57	0.50	0.012	< 0.01
c	0.20	0.20	0.006	0.83
PD	0.82	0.84	0.003	< 0.01
ED	0.68	0.72	0.006	< 0.01
Crude protein-degradation parameters				
a	0.17	0.37	0.032	< 0.01
b	0.75	0.55	0.03	< 0.01
c	0.22	0.22	0.006	0.73
PD	0.91	0.92	0.002	0.03
ED	0.75	0.80	0.008	< 0.01
Neutral detergent fiber-degradation parameters				
a	0.03	0.16	0.037	0.05
b	0.52	0.41	0.037	0.22
c	0.13	0.15	0.009	0.23
PD	0.55	0.57	0.020	0.82
ED	0.39	0.46	0.023	0.23

RC: raw rapeseed cake; FRC: fermented rapeseed cake; Significant differences were accepted at P < 0.05; SEM, standard error of the mean.

a a = soluble fraction; b = slowly degradable fraction; c = fractional rate of disappearance of the fraction b (/ h).

b PD = potential degradability,

c ED = effective degradability.

## 4 Discussion

FRC has a high concentration of CP (386 g/kg DM in FRC) and a low antinutritional factor content (i.e., glucosinolates, 1.66 μmol/g; ten times lower than in the raw RC) (Gao et al., 2020). The initial Hohenheim gas production study showed that most of the basic ruminal characteristics and total gas production were affected by FRC. The pH value, an essential parameter of rumen metabolism, increased in the FRC group to some extent because the FRC has lower sucrose content than RC (3.04% vs. 6.41% on a DM basis, respectively). Gao and Oba (2016) suggested that this may be directly related to the increase in ruminal pH. Higher ammonia concentration was associated with higher CP content in FRC, while the higher IVDMD and IVOMD was associated with the process of fermentation of FRC itself. For instance, the higher IVDMD and IVOMD for FRC could be partly attributed to the presence of less phytate-phosphorus and more available P due to the phytase involved in SSF (Allam et al., 2014). Another possible explanation for this is that RC contains higher oligosaccharides (raffinose and stachyose) known to reduce the digestion of diets (Selmi et al., 2013). The increase in IVDMD in the FRC group was also associated with an increase in CP, leading to elevated total degradable DM fractions (Chen et al., 2018). Moreover, the molar proportion of propionate increased in FRC treatment, on the contrary, the molar proportion of acetate, isobutyrate, and isovalerate decreased. A comparison of the findings with those of other studies confirms that the substitution of fermented soybean meal for unfermented soybean meal at 100% significantly increased the molar ratio of propionate in the rumen fluid of lactating Holstein calves (Wang et al., 2021). Moreover, Feizi et al. (2020) found that the fermented soybean meal at 9% and 13.5% of diets significantly lowered the molar ratio of acetate in the rumen fluid in Holstein calves. An increase in ammonia concentration and IVDMD and IVOMD was demonstrated with a simultaneous reduction in gas and CH_4_ production. The number of methanogens decreased, indicating the direct inhibition of CH_4_ production in the FRC group, but the mechanism is unclear. The SSF of RC caused a reduction in glucosinolates content (Drażbo et al., 2019) that might be converted to isothiocyanates, thiocyanates, nitriles, allylamine, benzylamine, and indole compounds (Aires et al., 2009). Sulfur containing isothiocyanates decreased CH_4_ in vitro (Lila et al., 2003, Soliva et al., 2011). Garlic oil containing different sulfur-containing compounds lowered CH_4_ production (Patra and Yu, 2012). In a previous study (Gao et al., 2020), the FRC, which is the same as the one used in the present study, had lower methionine and sodium sulfate than RC, which could suggest that more sulfur is released in FRC. As per Shah et al. (2020), if sulfur is fed to animals in the form of sulfate or the concentration of sulfur in the diet is higher, microorganisms in the rumen reduce sulfate to sulfides using hydrogen, increasing hydrogen sulfide levels in the rumen.

Ruminants have the ability to digest phytate-phosphorus through the action of phytase-producing bacteria residing anaerobically in the rumen. However, the process is not fully efficient (Mickdam et al., 2017). It has previously been observed that phytate-phosphorus decreased by 94.8% in the FRC product compared to RC (Drażbo et al., 2019). Therefore, FRC may have more P available. A comparison of the findings with Harder et al. (2015) confirms that raising P availability decreases total methanogens and *Methanosphaera* spp. in ruminal fluid. Besides, the improved nutrient digestibility and promotion of propionate formation during FRC incubation may also explain the decrease in ruminal CH_4_ production (Hristov et al., 2011).

Additionally, the Hohenheim syringe study showed a decrease in total gas production by 6% compared to the RC. The reduction in total gas production may also be explained by decreased CH_4_ production and lower levels of oligosaccharides, including raffinose and stachyose, that produce gas (Santoso et al., 2003). Previous studies have reported lower levels (22%) of oligosaccharides in FRC than in RC (Gao et al., 2020). Based on the favorable ruminal fermentation characteristics (including high pH and digestibility and lower CH_4_ production) of FRC, we used FRC as a dietary component for further in vitro and in vivo investigations.

The batch culture in vitro study and the in vivo study using cannulated dairy cows, in which FRC was used as the primary dietary protein source (FRC100), mostly confirmed the differences observed in the Hohenheim gas test. As with the Hohenheim experiment, an increase in the concentration of ammonia and a reduction in the total gas production, CH_4_ production, and number of methanogens were noted in the FRC diet. Surprisingly, the CH_4_ production and molar ration of propionate was found to have cubic effects in the batch culture study. Consequently, CH_4_ production related to other parameters (e.g., CH_4_/IVDMD, mmole/g) and TVFA production exhibited cubic effects, respectively. Additionally, in the batch culture and cannulated dairy cow experiments, an improvement in the digestibility of the diet and an increase in the concentration of propionate in the groups with FRC were confirmed. The main difference observed was the decreased pH value in the FRC100 group in the cannulated dairy cow study. The reduction in the pH value was likely due to the increase in *Lactobacillus* spp. as these bacteria are directly responsible for lactic acid production, which lowers pH in the rumen (Nagaraja and Titgemeyer, 2007, Patra and Kar, 2021). On the other hand, the increase in abundance of *Megasphaera elsdenii* in the FRC100 group may indicate that lactic acid stimulated the growth of this species of bacteria, thus maintaining relatively stable conditions in the rumen (mean pH = 6.04 for the FRC100 group; Chen et al., 2019). The FRC100 diet differed from the CONRC diet in the preliminary RC fermentation process. This process was carried out in SSF by a commercial enzyme expressed by *Pichia pastoris*. There are only a limited number of publications studying fermented products in the feeding of dairy cows. Feizi et al. (2020) showed a reduction of H_2_ producer *B. fibrisolvens* and *R. albus* abundance in Holstein calves fed fermented soybean meal. This agrees with the current finding where CH_4_ might decrease due to a limited H_2_ source. The lower abundance of *B. fibrisolvens* and *R. albus* may also explain the reduction in acetate observed in the batch culture and cannulated dairy cow experiments. On the other hand, the increased growth of *R. flavefaciens*, one of the most important cellulolytic bacteria, indicates that the effect of FRC100 on the rumen microbial population is inconclusive. In addition, an increase in the proportion of propionate was found, which was confirmed by an increase in the population of *Streptococcus bovis* and *Lactobacillus* spp., which are partly responsible for the transformation of nonstructural carbohydrates. *S. bovis* is the well-known amylolytic bacteria in the rumen, rapidly growing and producing mainly lactate with a limited amount of VFA (Chen and Wang, 2016). The lack of significant rumen acidification and changes in total VFA might be explained by the increase in *Megasphaera elsdenii*, which utilize lactic acid, thus protecting other rumen microorganisms against the adverse effects of low pH resulting from the accumulation of lactic acid (Chen et al., 2019). The higher ammonia concentration might be explained by increased protein degradation in FRC. The use of FRC100 lowered CH_4_ emission by 10% in the batch culture experiment (115 g/kg diet) and also in the in vivo experiment by 14% in exp. 3) 11% in exp. 4. The lower number of protozoa, as a result of the relationship between protozoa and methanogens (a symbiosis on which 37% of CH_4_ production depends; Newbold et al., 2015) can decrease the amount of CH_4_ production. Other mechanisms such as the inhibition of rumen methanogenesis as a result of an increase in the propionate concentration, or through direct inhibition of the growth of methanogens may decrease CH_4_ (Puchalska et al., 2021). Notably, the reduction in CH_4_ emissions was not linked to a decrease in digestibility or changes in the primary composition of milk. The only significant difference was the increase in the concentration of urea in milk, which may be due to greater concentration of ammonia in the rumen fluid.

It has previously been observed that dietary rapeseed products, such as rapeseed meal, cake, and rapeseed oil, can enhance levels of UFA, including MUFA and PUFA, in the milk of dairy cows (Givens et al., 2009, Hristov et al., 2011). The fatty acid profile of milk depends on the content of dietary fat, its transformation in the rumen, and de novo synthesis in the mammary gland. In the present study, the total fat content of the diets was 26 g/kg DM and 25 g/kg DM for RC and FRC, respectively and these account for, accordingly, 603 g/d/cow and 580 g/d/cow.

Our research showed that the proportions of most FA in the ruminal fluid and milk did not fully mimic those of the diet. Most proportions were not affected by FRC treatment in the Hohenheim test experiment (Table 3), with the exceptions of C16:0, which increased in the ruminal fluid after in vitro fermentation, and of C18:0, which decreased. The lower proportion of C18:0 in the ruminal fluid in the FRC group may result from the ruminal microbial biohydrogenation of C18 UFA (McNamee et al., 2002).

The batch culture experiment also resulted in a lower concentration of C18:0 in the FRC diets (quadratic and cubic contrasts) and a higher level of C18:2 and these changes were dose-dependent. Higher dietary FRC resulted in a smaller decrease in C18:0 and a minor increase in C18:2 *cis*-9 *cis*-12. In the present study, RC and FRC contained similar fat content (100 g/kg DM in RC vs. 95 g/kg DM in FRC, respectively), which seems unlikely to modulate the FA proportion in the rumen due to the fat content difference. It may be related to ruminal biohydrogenation directly. No linear and cubic effect of FRC was noted on *cis*-9 *trans*-11 C18:2, whereas *trans*-10 *cis*-12 C18:2 decreased in the diet with 50, 75%, and 100% FRC levels. The results suggest that diets with FRC mitigated the first step of biohydrogenation. Usually, up to 95% of C18:2 *cis*-9 *cis*-12 ruminal biohydrogenation is noted (Bauman et al., 2003). The increase in C18:3 *cis*-9 *cis*-12 *cis*-15 was dose-dependent; however, C18:1 *trans-*11, one of the intermediary products of C18:3 biohydrogenation, decreased, which also could reflect that the initial step of biohydrogenation was limited (Dewanckele et al., 2020).

The changes in C18:1 and C18:2 levels seen in the batch culture and the cannulated dairy cow experiments could be the indicators of complete rumen biohydrogenation without the stages of isomerization when conjugated isomers are formed since C18:1 *cis*-9 and C18:2 *cis*-9 *cis*-12 were the main FAs present in FRC. Similar results were obtained by Goiri et al. (2021) in Holstein and Brown Swiss dairy cows (172 ± 112 d in milk) receiving 1.64 kg of cold-pressed rapeseed cake/d/cow. A higher proportion of ruminal C18:0 was found in dairy cows fed cold-pressed rapeseed cake, which, like the FRC, provided greater amounts of C18 UFAs than the control diet. Benhissi et al. (2016) and Zubiria et al. (2019) also observed the same trends.

The FRC diets in the batch culture system led to decreased SFA and increased UFA (mostly MUFA), which were also confirmed in the rumen-cannulated dairy cow experiment. C18:1 *trans*-11 and *trans*-10 *cis*-12 C18:2 concentrations were lower in the FRC of batch culture trial than in the CONRC group, which agrees with the results of Shingfield and Griinari (2007), who mentioned *trans*-11 18:1 as an intermediate of ruminal linoleic acid metabolism derived from the reduction of *trans*-10 *cis*-12 conjugated linoleic acid. Lower *trans*-10 *cis*-12 conjugated linoleic acid in the FRC groups decreased *trans*-11 18:1 content in ruminal fluid. These results were not confirmed in the cannulated dairy cow experiment.

The in vivo cannulated dairy cow study results confirmed that FRC limited ruminal C18:2 *cis-*9 *cis-*12 isomerization and C18:2 *cis-*9 *trans-*11 biohydrogenation to C18:1 *trans*-11 and C18:0 (AbuGhazaleh et al., 2002). It is important to highlight that the final reduction step of C18:2 is considered to be rate-limiting, and C18:1 intermediates (mostly C18:1 *trans*-11) can thus accumulate and generally flow out of the rumen when excessive amounts of UFA are ingested (Bessa et al., 2007, Shingfield et al., 2013, Goiri et al., 2021). Compared with the control diet, this effect might be an alteration in the population of ruminal microorganisms by FRC diets. In this study, we did not evaluate the direct impact of the biohydrogenation of bacteria. Higher C18:1 *cis*-9 concentrations were observed in the ruminal fluid for the FRC diets. Benhissi et al. (2016) found similar C18:1 *cis*-9 concentrations in the ruminal fluid when cold-pressed rapeseed cake was included in the diet (148 g/kg on a DM basis). The FRC tended to decrease SFA with the decrease in the most abundant SFA, C16:0 and C18:0.

In the milk, the FRC diet significantly increased milk conjugated C18:2 isomers (C18:2 *cis*-9 *trans*-11 and C18:2 *trans*-10 *cis*-12) and n-3 PUFA content by 7.84%, which are considered healthier for humans. C18:1 *trans*-11 concentration tended to increase with the FRC diet. AbuGhazaleh et al. (2002) suggested that increasing milk C18:1 *trans*-11 could elevate *cis*-9 *trans*-11 via the delta-9 desaturase reaction in the mammary gland. FRC fed to dairy cows under a commercial production system improved the n-6 to n-3 FA ratio. The current study found a tendency to increase mRNA expression of the *ELOVL5* gene. The higher transcript content of the *ELOVL5* gene is associated with higher content of C14:0 (Zhu et al., 2017), C18:0, and C18:1 (Green et al., 2010, Matsumoto et al., 2013), and n-3 FA (Cherfaoui et al., 2012).

The FeedExpert software was used to calculate the feeding costs of dairy cows based on the cost of particular feed components. The calculations considered the FRC price (€0.2/kg RC vs. €0.7/kg FRC) given by the HiProMine company. The cost of RC feed for the CONRC group was €0.59/d/cow, whereas the cost of FRC feed for the FRC100 group was €1.92/d/cow. The results from experiment 3 indicated that FRC can reduce CH_4_ emissions. However, the cost of FRC was 0.052 euros per kg of milk and the price of RC was only 0.016 euros. The price for FRC is three times higher, and it is, therefore, less likely that farmers would use FRC to mitigate CH_4_ under the current situation, but a large-scale production of FRC using high-throughput technology might reduce the cost in future.

## 5 Conclusion

Feeding FRC at 11.5% of the diet to dairy cows did not affect DMI, milk production, or composition but improved the milk FA profile by increasing the concentrations of C18:2 *cis*-9 *trans*-11, C18:2 *trans*-10 *cis*-12, and MUFA and decreasing the ratio of n-6 to n-3 FA. Also, feeding FRC at 11.5% of the diet to dairy cows can effectively mitigate ruminal CH_4_ production. Thus, FRC may be used in the diet of lactating cow to lower CH_4_ production and improve milk FA profile without adversely affecting ruminal fermentation, nutrient utilization and milk production.

## Funding

This study was supported by a grant from Poznań University of Life Sciences, Research grant for young researchers, Poland (Grant No. 506.533.04); European Union’s Horizon 2020 Research and Innovation Program under Grant Agreement No 696356 for research carried out within the ERA-GAS/ERA-NET SUSAN/ICT-AGRI project CCCfarming (SUSAN/II/CCCFARMING/03/202); National Science Center, Poland (Grant No. 267659/7/NCBR/2015). The publication was co-financed within the framework of the Polish Ministry of Science and Higher Education’s Program: “Regional Initiative Excellence” in the years 2019–2022 (No. 005/RID/2018/19) “financing amount 12 000 000,00 PLN”.

## CRediT authorship contribution statement

**Min Gao**: Conceptualization, Methodology, Writing – original draft. **Adam Cieslak**: Investigation, Data curation, Writing – review & editing. **Haihao Huang**: Data curation. **Maciej Gogulski**: Methodology, Data curation. **Diana Ruska**: Data curation. **Daniel Petric**: Investigation. **Amlan Kumar Patra**: Writing – review & editing. **Mohamed El-Sherbiny:** Writing - review. **Malgorzata Szumacher-Strabel**: Supervision, Data curation, Writing – review & editing.

## Declaration of Competing Interest

The authors have no conflict of interest to declare for the manuscript entitled “Effects of crude and fermented rapeseed cake on ruminal fermentation, methane emission, and milk production in lactating dairy cows” by Min Gao, Adam Cieślak, Haihao Huang, Maciej Gogulski, Daniel Petric, Diana Ruska, Amlan Kumar Patra, and Małgorzata Szumacher-Strabel, that we submit for publication in Animal Feed Science and Technology journal.
