# Supplementary material for: Effects of raw and fermented rapeseed cake on ruminal fermentation, methane emission, and milk production in lactating dairy cows
Source: Anim Feed Sci Technol. 2023 Jun;300:115644. doi: 10.1016/j.anifeedsci.2023.115644 (PMC10204938; doi:10.1016/j.anifeedsci.2023.115644)
Supplement: Supplementary file 1 — Supplementary material [file mmc1.docx]

STable 1. The sequences of primers specific to the analyzed expression of six genes [*acetyl-CoA carboxylase 1* (*ACACA*), *stearoyl-CoA desaturase* (*SCD*), *fatty acid elongase 5* (*ELOVL5*), *fatty acid synthase* (*FASN*), *fatty acid desaturase 1* (*FADS1*), and *lipoprotein lipase* (*LPL*)] in the somatic cells of milk of lactating cows.

| Gene name | Primer sequence (5' to 3')* | Reference |
| --- | --- | --- |
| *ACACA* | F: “CATCTTGTCCGAAACGTCGAT“ | Abdelatty, A.M et al., 2017 |
|  | R: “CCCTTCGAACATACACCTCCA“ |  |
| *SCD* | F: “TCCTGTTGTTGTGCTTCATCC“ | Abdelatty, A.M et al., 2017 |
|  | R: “GGCATAACGGAATAAGGTGGC“ |  |
| *ELOVL5* | F:“TGCTTCAGTTTGTGCTGACC“ | Urrutia et al., 2015 |
|  | R “TGGTCCTTCTGGTGCTCTCT“ |  |
| *FASN* | F: “ACCTCGTGAAGGCTGTGACTCA“ | Abdelatty, A.M et al., 2017 |
|  | R: “TGAGTCGAGGCCAAGGTCTGAA“ |  |
| *FADS1* | F: ”CTGCTGTACCTGCTGCACAT“ | Urrutia et al., 2015 |
|  | R: ”ACGGACAGGTGTCCAAAGTC” |  |
| *LPL* | F: ”ACACAGCTGAGGACACTTGCC“ | Abdelatty, A.M et al., 2017 |
|  | R: ”GCCATGGATCACCACAAAGG” |  |

* Primer direction (F: forward; R: reverse).

STable 2. The sequences of primers specific to the analyzed bacteria species.

| Species | Primer sequence (5' to 3')* | Reference |
| --- | --- | --- |
| *Ruminococcus flavefaciens* | F:“CGAACGGAGATAATTTGAGTTTACTTAGG“ | Zeng et al., 2012 |
|  | R: “CGGTCTCTGTATGTTATGAGGTATTACC“ |  |
| *Fibrobacter succinogenes* | F: “GTTCGGAATTACTGGGCGTAAA“ | Denman et al., 2006 |
|  | R: “CGCCTGCCCCTGAACTATC“ |  |
| *Streptococcus bovis* | F: ”TTCCTAGAGATAGGAAGTTTCTTCGG“ | Yu et al., 2005 |
|  | R: ”ATGATGGCAACTAACAATAGGGGT” |  |
| *Prevotella* spp. | F: ”GAAGGTCCCCCACATTG “ | Yu et al., 2005 |
|  | R:”CAATCGGAGTTCTTCGTG” |  |
| *Butyrivibrio proteoclasticus* | F: “TCCTAGTGTAGCGGTGAAATG“ | Potu et al., 2011 |
|  | R: “TTAGCGACGGCACTGAATGCCTA“ |  |
| *Ruminococcus albus* | F:“CCCTAAAAGCAGTCTTAGTTCG“ | Wang et al., 1997 |
|  | R “CCTCCTTGCGGTTAGAACA“ |  |
| *Butyrivibrio fibrisolvens* | F: “ACACACCGCCCGTCACA“ | Li et al., 2009 |
|  | R: “TCCTTACGGTTGGGTCACAGA“ |  |
| *Lactobacillus* spp. | F: ”TATGGTAATTGTGTGNCAGCMGCCGCGGTAA“ | Poeker et al., 2018 |
|  | R: ”AGTCAGTCAGCCGGACTACHVGGGTWTCTAAT” |  |
| *Megasphaera elsdenii* | F: ”AGATGGGGACAACAGCTGGA“ | Yu et al., 2005 |
|  | R: ”CGAAAGCTCCGAAGAGCCT” |  |

* Primer direction (F: forward; R: reverse).

Abdelatty, A.M., Iwaniuk, M.E., Garcia, M., Moyes, K.M., Teter, B.B., Delmonte, P., Kadegowda, A.K.G., Tony, M.A., Mohamad, F.F., Erdman, R.A., 2017. Effect of short-term feed restriction on temporal changes in milk components and mammary lipogenic gene expression in mid-lactation Holstein dairy cows. J. Dairy Sci. 100, 4000–4013. https://doi.org/10.3168/jds.2016-11130.

Denman, S.E., McSweeney, C.S., 2006. Development of a real-time PCR assay for monitoring anaerobic fungal and cellulolytic bacterial populations within the rumen. FEMS Microbiol. Ecol. 58, 572–582. https://doi.org/10.1111/j.1574-6941.2006.00190.x.

Li, M., Penner, G.B., Hernandez-Sanabria, E., Oba, M., Guan, L.L., 2009. Effects of sampling location and time, and host animal on assessment of bacterial diversity and fermentation parameters in the bovine rumen. J. Appl. Microbiol. 107, 1924–1934. https://doi.org/10.1111/j.1365-2672.2009.04376.x.

Poeker, S.A., Geirnaert, A., Berchtold, L., Greppi, A., Krych, L., Steinert, R.E., De Wouters, T., Lacroix, C., 2018. Understanding the prebiotic potential of different dietary fibers using an in vitro continuous adult fermentation model (PolyFermS). Sci. Rep. 8, 1–12. https://doi.org/10.1038/s41598-018-22438-y.

Potu, R.B., AbuGhazaleh, A.A., Hastings, D., Jones, K., Ibrahim, S.A., 2011. The effect of lipid supplements on ruminal bacteria in continuous culture fermenters varies with the fatty acid composition. J. Microbiol. 49, 216–223. https://doi.org/10.1007/s12275-011-0365-1.

Urrutia, O., Soret, B., Insausti, K., Mendizabal, J.A., Purroy, A., Arana, A., 2015. The effects of linseed or chia seed dietary supplementation on adipose tissue development, fatty acid composition, and lipogenic gene expression in lambs. Small Rumin. Res. 123, 204–211. https://doi.org/10.1016/j.smallrumres.2014.12.008.

Wang, R.F., Cao, W.W., Cerniglia, C.E., 1997. PCR detection of Ruminococcus spp. in human and animal faecal samples. Mol. Cell. Probes 11, 259–265. https://doi.org/10.1006/mcpr.1997.0111.

Yu, Y., Lee, C., Kim, J., Hwang, S., 2005. Group-specific primer and probe sets to detect methanogenic communities using quantitative real-time polymerase chain reaction. Biotechnol. Bioeng. 89, 670–679. https://doi.org/10.1002/bit.20347.

Zeng, J., Bian, Y., Xing, P., Wu, Q.L., 2012. Macrophyte species drive the variation of bacterioplankton community composition in a shallow freshwater lake. Appl. Environ. Microbiol. 78, 177–184. https://doi.org/10.1128/AEM.05117-11.
